# Metabolomic Profiling of Medicinal Plant-Based Soft Porridge and Evaluation of Cytotoxicity

**DOI:** 10.3390/metabo16090606

**Published:** 2026-08-25

**Authors:** Phato Avheani Matsheketsheke, Nakisani Babra Moyo, Bono Nethathe

**Affiliations:** Department of Food Science and Technology, Faculty of Science, Engineering and Agriculture, University of Venda, Private Bag X5050, Thohoyandou 0950, South Africa; 15018368@mvula.univen.ac.za (P.A.M.); bono.nethathe@univen.ac.za (B.N.)

**Keywords:** infants’ food, medicinal plants, secondary metabolites, UHPLC-qTOF-MS, toxicity, Venda ethnomedicine

## Abstract

**Background/Objectives:** Traditional medicinal plants play a crucial role in infants’ healthcare in underdeveloped countries and communities where access to modern medicine is limited. In Limpopo, the Vhavenda tribe has a custom of feeding babies as young as a day old with *tshiunza*, a very light and warm, soft porridge prepared in medicinal plant extracts, believed to help treat various ailments and boost the immune and digestive systems. However, reports of negative side effects, including vomiting, haematuria, renal inflammation and hospitalisations, highlight safety concerns of these traditional practices. This study aimed to profile the chemical composition of soft porridge samples prepared in aqueous extracts of medicinal plants reported to be commonly used in infants’ food in the Venda region of Limpopo; *Annona senegalensis* (roots), *Piliostigma thonningii* (leaves), *Carissa edulis* (roots) and *Bauhinia galpinii* (leaves). **Methods:** Ultrahigh-performance liquid chromatography coupled to quadrupole time-of-flight mass spectrometry (UHPLC-qTOF-MS) was used for metabolic profiling, followed by putative compound identification using feature-based molecular networking and a compound fragmentation predictor tool, SIRIUS. To evaluate the toxicity of these plants, a cytotoxicity assay was conducted using plant extracts on African monkey kidney cells. **Results:** Different classes of polyphenols, including flavonoids (flavan-3-ols and flavonols) and hydroxycinnamoyl amides, were among the dominant compounds putatively identified in the methanolic extracts of soft porridge prepared from the four plant extracts. *A. senegalensis* aqueous extracts were inactive (IC_50_ > 500 µg/mL, 55.57% viability at 500 µg/mL), while *P. thonningii* was non-cytotoxic (105–132% viability within the tested concentration range). **Conclusions:** Although no toxic compounds were identified and no cytotoxic potential was observed in the tested plant extracts, this does not translate to the safe use of medicinal plants in infants’ food. Therefore, future studies should conduct pharmacological and toxicological evaluations to better understand the potential risks of these plant extracts to infant health.

## 1. Introduction

Medicinal plants have long served as an important source of therapeutic compounds, with rural communities utilising their bioactive properties to manage a wide range of ailments [1]. In recent years, interest has grown in incorporating such botanicals into infant foods due to their perceived health and nutritional benefits [2]. Extracts from different medicinal plant parts are commonly used in low-resourced communities across various countries to prepare soft porridge and infant formula for babies as young as one month old [3]. In Limpopo Province, South Africa, some commonly used plants in the preparation of soft porridge (*tshiunza*) include *Annona senegalensis*, *Bauhinia galpinii*, *Piliostigma thonningii* and *Carissa edulis*. Traditionally, *A. senegalensis* is administered to infants to improve digestion and manage diarrhoea, dysentery, and delayed fontanelle closure [4]. Infusions prepared from *B. galpinii* leaves are commonly used to alleviate gastrointestinal discomfort [5,6,7]. On the other hand, *C. edulis* roots and leaves are used in herbal preparations intended to strengthen immunity and treat coughs and other respiratory conditions in young children [8]. In several African communities, decoctions and infusions of *P. thonningii* are incorporated into soft porridge or herbal drinks traditionally consumed to support nourishment and growth in infants and young children [9,10,11].

Despite their widespread use, concerns have been raised regarding the safety of administering medicinal plant-based food to infants. The World Health Organisation advises against the use of such treatments in infants younger than six months [12]. Reports have associated the consumption of medicinal plant-based infant food with adverse effects, including vomiting, haematuria, urinary retention, and, in severe cases, hospitalisation. Clinical observations have also indicated signs of renal inflammation in affected infants [13]. These findings underscore the need for a critical evaluation of the safety of medicinal plant use in early-life nutrition.

Infants may be particularly vulnerable to plant-derived compounds due to their immature metabolic systems. The activity of cytochrome P450 enzymes in infants is less, operating at approximately 30–50% of adult capacity [14]. In addition, underdeveloped glucuronidation pathways can prolong the half-lives of xenobiotics, including plant secondary metabolites [15]. The increased permeability of the blood–brain barrier in early life may further allow neurotoxic compounds to access developing neural tissue [16]. Together, these physiological limitations may enhance the risk of accumulation and toxicity of plant-derived metabolites in infants.

The reported adverse effects on infants fed food containing medicinal plant extracts might be attributed to toxic compounds contained in these plants, inappropriate dosages administered to infants and immature neonatal metabolic systems. Therefore, metabolic profiling can serve as a fundamental step towards understanding the safety of these plants. Compound characterisation through high-resolution mass spectrometry instruments is essential for downstream *in vitro* and *in vivo* toxicological studies of bioactive or toxic molecules [17], although metabolomic profiling on its own cannot ascertain the safety of detected compounds. The chemical composition of most plants reported to be used in infant feeding practices or infants’ food formulated from these plants is currently not fully known. Most studies that have investigated these plants have focused on total phenolic compounds and antioxidant activities or have identified a few compounds [5,18].

To the best of our knowledge, this is the first study that integrated metabolite profiling, feature-based molecular networking and preliminary cytotoxicity assessment of medicinal plant-based infant foods. The aim of this study was to comprehensively characterise the chemical composition of soft porridge prepared using aqueous extracts of *A. senegalensis*, *B. galpinii*, *P. thonningii* and *C. edulis*, traditionally fed to infants. The UHPLC-qTOF-MS was used for metabolite profiling followed by compound annotation using feature-based molecular networking and SIRIUS, a fragmentation prediction tool. Molecular networking further enabled comparison of metabolite profiles between plant extracts and the corresponding soft porridge samples, allowing assessment of metabolite persistence following cooking. Preliminary toxicity of selected plant extracts was evaluated using the 3-[4,5-dimethylthiazol-2-yl]-2,5-diphenyl tetrazolium bromide (MTT) cytotoxicity assay on selected plant extracts.

## 2. Materials and Methods

### 2.1. Metabolic Profiling of Selected Plants Reported to Be Used in Infants’ Food Using the UHPLC-qTOF-MS

#### 2.1.1. Chemicals and Reagents

LC-MS grade acetonitrile, methanol and water were supplied by Romil SpS (Cambridge, UK), while analytical-grade formic acid was sourced from Sigma-Aldrich (Johannesburg, South Africa), for the plants’ metabolic profiling. For the MTT assay, Dulbecco’s Modified Eagle Medium (DMEM), Minimal Essential Medium (MEM), RPMI and PBS with and without Ca^2+^ and Mg^2+^ were purchased from Cytiva (Marlborough, MA, USA). Foetal Bovine Serum (FBS) and penicillin/streptomycin were purchased from Biowest (Nuaillè, France). African green monkey kidney epithelial (Vero) cell lines were purchased from Cellonex (Pty) Ltd., Johannesburg, South Africa.

#### 2.1.2. Plant Collection

Plant materials were collected from Tshakhuma, Ha-Masia and the University of Venda in Limpopo, Venda. The plants used in the study were *A. senegalensis* (roots), *C. edulis* (roots), *B. galpinii* (leaves) and *P. thonningii* (leaves). Before using these plant materials in the laboratory, a qualified botanist, Prof MP Tshisikhawe from the Department of Biological Sciences at the University of Venda, confirmed their identification and the following voucher specimens (MPT00170, MPT00202, MPT00143 and MPT00188) were issued for *A. senegalensis*, *C. edulis*, *B. galpinii*, and *P. thonningii*, respectively.

a. Sample preparation
  i. Preparation of plant materials

The soft porridge (*tshiunza*) was prepared according to the methods described by the infant caregivers and the traditional healers, with modifications [4]. Fresh roots of *A. senegalensis* and *C. edulis* and fresh leaves of *B. galpinii* and *P. thonningii* were washed with distilled water, after which 100 g of each plant material was boiled in 600 mL of distilled water for 10 min. The resulting aqueous extracts were used to prepare the soft porridge samples. The plant extracts were strained, followed by the addition of 100 g of maize meal to the boiling plant extracts (500 mL) and cooked for 15 min for the preparation of soft porridge samples. The negative control was plain soft porridge prepared from 100 g of maize meal and 500 mL of boiling distilled water, also cooked for 15 min. All the prepared soft porridge samples were spread on a tray and air-dried to complete dryness. Thereafter, all soft porridge samples were ground into fine powder for further analysis. All samples were prepared in duplicates. For the positive control, washed roots of *A. senegalensis* and *C. edulis* and washed leaves of *B. galpinii* and *P. thonningii* were air-dried and ground. For *A. senegalensis*, plant powder was also added to cooked plain soft porridge, as findings from surveys suggested this as one of the preparation methods of this particular plant [4].

  ii. Secondary metabolites extraction from plant-based soft porridge

Metabolite extraction was performed according to the slightly modified method described in a previous study [19]. One (1) g of each plant-based soft porridge was extracted in 10 mL of 80% methanol on a digital rotisserie tube rotator (Dlab Scientific, Beijing, China) at 70 rpm overnight. The choice of 80% methanol as the extraction solvent was based on its ability to extract a broad range of polar and semi-polar metabolites, thereby providing wider metabolite coverage for LC-MS analysis. The resulting crude extracts were centrifuged on a Rotina 380r centrifuge (Labotec Ecotherm, Midrand, South Africa) at 2739× *g* for 15 min. The samples were then filtered through 0.22 µm Nylon filters into amber 2 mL HPLC vials before being injected into the UHPLC-qTOF-MS. Plain soft porridge (negative) control and plant powder (positive control) were also prepared according to this method.

  iii. Chromatographic separation

The soft porridge samples prepared in *A. senegalensis*, *C. edulis*, *B. galpinii* and *P. thonningii* extracts, together with the negative (plain soft porridge) and positive control (plant) sample methanolic extracts, were analysed using a liquid chromatography quadrupole time-of-flight tandem mass spectrometer (LC-MS-9030 q-TOF, Shimadzu Corporation, Kyoto, Japan) following a method previously used by Moyo et al. [20]. Secondary metabolites were separated using a Shim-pack Velox C_18_ column (100 × 2.1 mm, 2.7 µm) (Shimadzu Corporation, Kyoto, Japan) maintained at 55 °C. A volume of 3 µL was injected into the instrument and the compounds in the extracts were separated using a binary mobile phase gradient elution at a flow rate of 0.3 mL min^−1^. Mobile phases A and B were water and methanol, respectively, containing 0.1% formic acid. The chromatographic gradient started at 10% mobile phase B and was maintained at this composition for 2 min, followed by an increase to 60% B at 5 min. The composition was further increased to 90% B between 8 and 11 min. At 12 min, the mobile phase was reduced to 60% B, then further decreased to 10% B and held at this composition until 15 min. Mass spectral analysis was performed using a qTOF high-resolution mass spectrometer equipped with an electrospray ionisation (ESI) interface operating in negative ionisation mode. The mass spectrometric conditions were as follows: interface voltage, 4.0 kV; interface temperature, 300 °C; nebulising gas flow, 3 L min^−1^; dry gas flow, 3 L min^−1^; heat block temperature, 400 °C; DL temperature, 280 °C; detector voltage, 1.8 kV; and flight tube temperature, 42 °C. Sodium iodide was used as the calibration solution to ensure high mass accuracy. MS^1^ and MS^2^ spectra were acquired simultaneously using data-dependent acquisition for ions within an *m*/*z* range of 100–1000 Da at an intensity threshold of 5000. Argon was used as the collision gas during MS^2^ acquisition, at a collision energy of 30 eV with a spread of 5.

  iv. Feature-based molecular networking and in silico fragmentation pattern prediction

Feature-based molecular networking was done on the GNPS2 platform from data that was pre-processed using MS Dial version 4.9. Raw data in mzML format was uploaded to MS Dial, and mass accuracy for both MS^1^ and MS^2^ was set at ±0.02 Da. Peak detection was done according to MS Dial default settings (minimum peak height was 1000 amplitude and the mass slice at 0.1 Da). The detected features were aligned across samples with retention time and MS^1^ tolerances set at 0.05 min and 0.02 Da, respectively. Gap filling by compulsion was enabled [21]. For generating a feature-based molecular network, mass tolerances on GNPS2 for precursor and MS^2^ fragment ions were set at 0.02 Da. Spectral similarity was evaluated using a cosine score threshold of 0.6, with a minimum requirement of four matched fragment ions for inclusion in the molecular network. The Top K value was set to 25, while the maximum component size was limited to 50. For library matching, the cosine score was set at 0.7 with four matched fragment ions. The generated molecular network was visualised using Cytoscape version 3.10.3 [22]. Feature-based molecular networking was used for the visualisation of the chemical composition of the soft porridge samples and the corresponding plant samples.

SIRIUS version 6.2.2 was employed to predict fragmentation patterns of compounds of interest identified in this study [23]. Data in mgf format was imported into SIRIUS v.6.2.2 for analysis. For molecular formula prediction, negative ionisation mode was selected as the possible ionisation type, the MS/MS isotope scorer was set to “score”, and the permitted elements for molecular formula generation were carbon (C), hydrogen (H), potassium (K), phosphorus (P) and oxygen (O). Structural database searches were conducted against PubChem, GNPS, HMDB, KNApSAcK, COCONUT, ChEBI, and Natural Products databases. Compound class prediction was performed using CANOPUS. The mass error was set at 5 ppm. Only candidate structures with CSI:FingerID scores closer to zero were considered for tentative annotation. For compound class assignment, the highest-ranked CANOPUS prediction was reported when it was consistent with the molecular formula and the tentative structural annotation generated by SIRIUS.

b. MTT (3-[4,5-dimethylthiazol-2-yl]-2,5 diphenyl tetrazolium bromide) assay

For the MTT assay, only *A. senegalensis* and *P. thonningii* were selected for preliminary cytotoxicity analysis, guided by metabolic profiling results (most compounds persisted after cooking) and by ethnobotanical information from infants’ caregivers, elders, and traditional healers. Extracts were prepared according to a previous report with minor modifications [24]. The plants, ground to a fine powder, were macerated in a fivefold excess of water in extraction pots, with the solvent level above that of the plant material. The slurry was maintained at room temperature for 48 h, then centrifuged at 300 rpm for 5 min and filtered using filter paper with a pore size of 60 µm. The process was repeated twice, yielding a total of three extractions [25]. The combined extracts were concentrated in a freeze dryer and transferred to labelled vials. A 3–5 g sample of each plant extract was used for the cytotoxicity assay, and 3 g was kept in the extract bank for subsequent use. Stock solutions were prepared by dissolving the extracts in 10% dimethyl sulphoxide (DMSO). Thereafter, the extracts were solubilised in DMSO to yield a stock concentration of 100 mg mL^−1^ and stored at 4 °C until use.

  i. Cell line maintenance

The Vero cell line was originally established from the kidney epithelial tissue of the African green monkey (*Cercopithecus aethiops*). This cell line is characterised by a homozygous deletion on chromosome 12 that results in the loss of the type I interferon (IFN-α and IFN-β) gene cluster. This makes the cells deficient in interferon production but responsive to exogenous interferon. This genetic characteristic makes them a widely used mammalian cell model for assessing cytotoxicity sensitively [26]. Complete growth medium consisted of DMEM supplemented with 10% FBS and 1× penicillin–streptomycin for Vero cells. Cells were maintained in 10 cm culture dishes in complete medium and incubated at 37 °C in a humidified atmosphere with 5% CO_2_.

  ii. Screening protocol

The African monkey kidney cell line (Vero) was used as an appropriate first-line mammalian cytotoxicity model for screening plant extracts in ethnopharmacological studies. The cells were seeded into a 96-well microtiter plate at confluence (3 × 10^4^ cells/mL) using a volume of 100 μL in each well. The microtiter plates were incubated at 37 °C, 5% CO_2_, and 100% relative humidity for 24 h prior to the addition of test extracts to allow for cell attachment. Cells were treated with a 6-point serial dilution of 18.75, 37.5, 75, 150, 300, and 500 µg mL^−1^ of each extract. Melphalan was used as a positive control at 18.75 to 500 µg mL^−1^. Untreated cells were used as a negative control. Cells were incubated for 48 h. Treatments were aspirated, and 100 μL of MTT (0.5 mgmL^−1^) in complete medium was added to each well, which was incubated for 3 h. MTT was aspirated and 100 μL of DMSO was added to each well. Absorbance was measured at 540 nm using a BioTek^®^ PowerWave XS spectrophotometer (Winooski, VT, USA). The results were normalised to untreated control, defined as 100% cell viability, and were presented as the mean ± standard deviation from three independent experiments. Half-maximal inhibitory concentrations (IC_50_) were derived from 4-parameter logistic regression. Statistical analyses were conducted in Python version 3.13.14 using SciPy and statistical significance was defined as *p* < 0.05.

## 3. Results

### 3.1. Profiling of Soft Porridge Samples Prepared in Aqueous Extracts of Selected Plants Reported to Be Used in Infants’ Food

**Figure 1:**
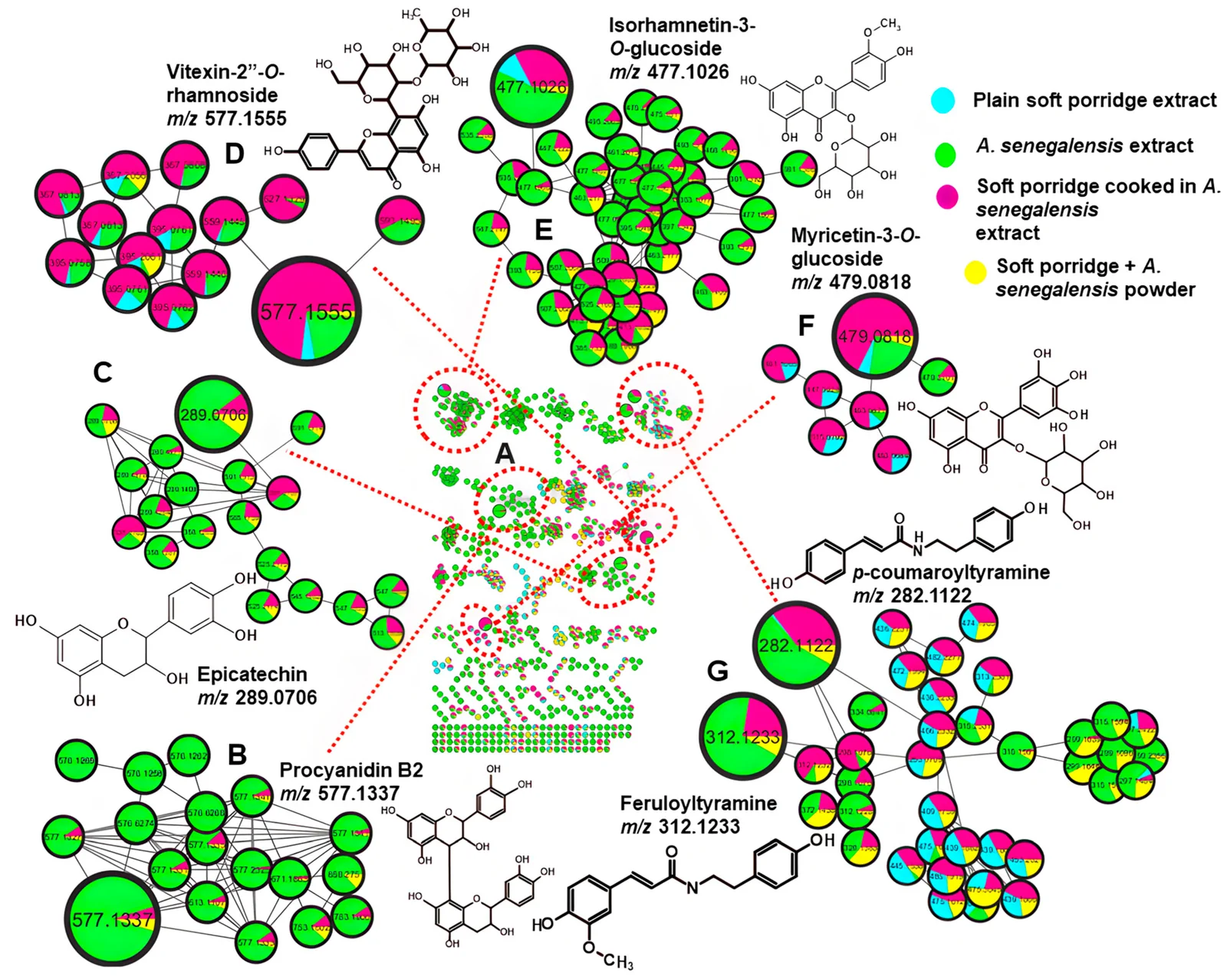
Visual presentation of the metabolic profile of soft porridge prepared in *A. senegalensis* extract using the UHPLC-qTOF-MS and feature-based molecular networking, with (**A**) showing the full molecular network, (**B**–**F**) different flavonoid subclasses and (**G**) hydroxycinnamoyl amides. Each node represents a feature, and the different colours represent the samples in which they were observed. All the compounds with enlarged nodes were observed in soft porridge prepared in the plant extract (pink colour in the nodes).

**Figure 2:**
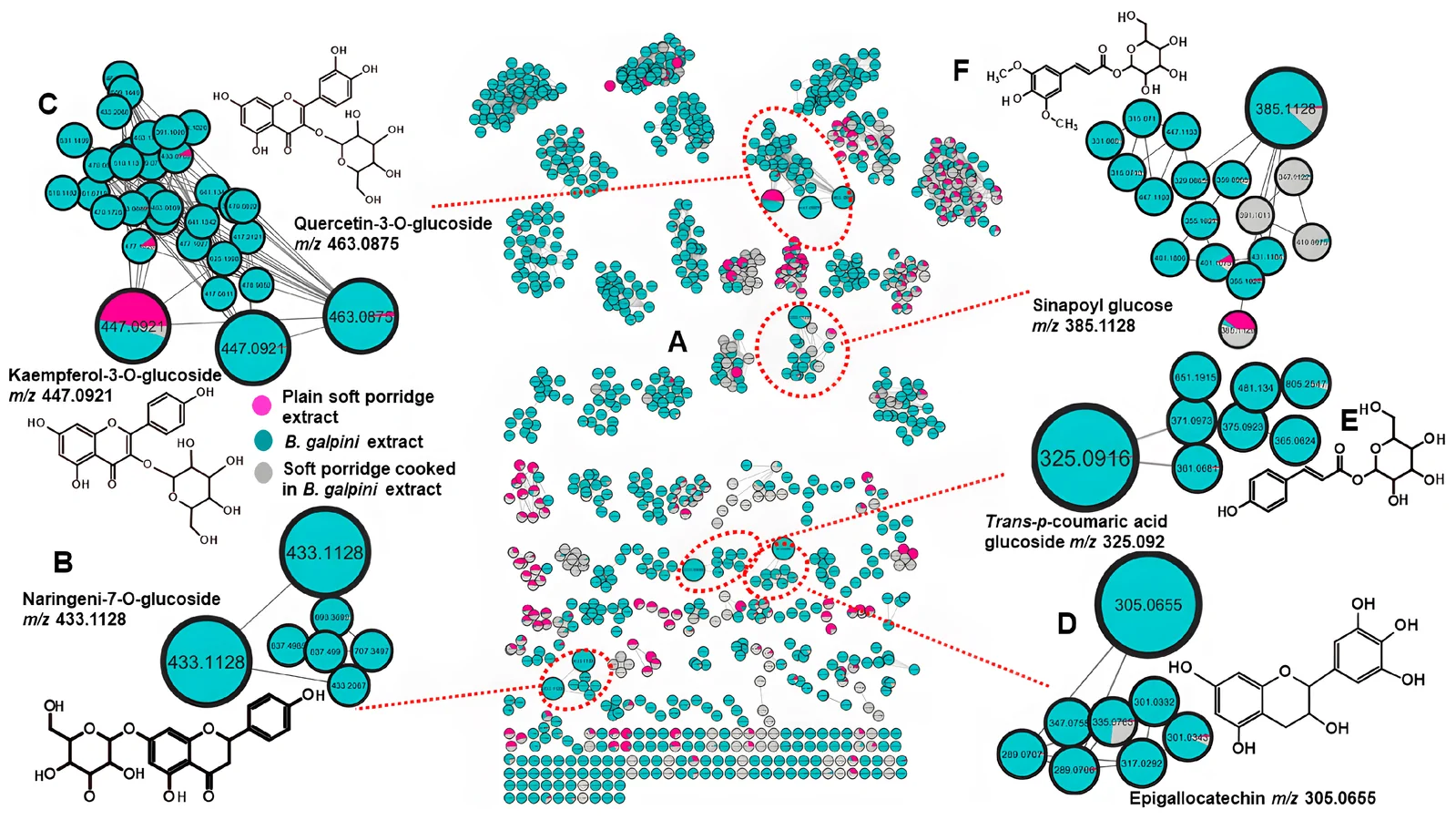
Infographic presentation of the metabolic profile of soft porridge prepared in *B. galpinii* extract (grey) using the UHPLC-qTOF-MS and feature-based molecular networking. Methanolic extracts of plain soft porridge and the plant are displayed in pink and blue, respectively. (**A**) Full network, (**B**–**D**) different flavonoid subclasses and (**E**,**F**) hydroxycinnamoyl glucosides.

**Figure 3:**
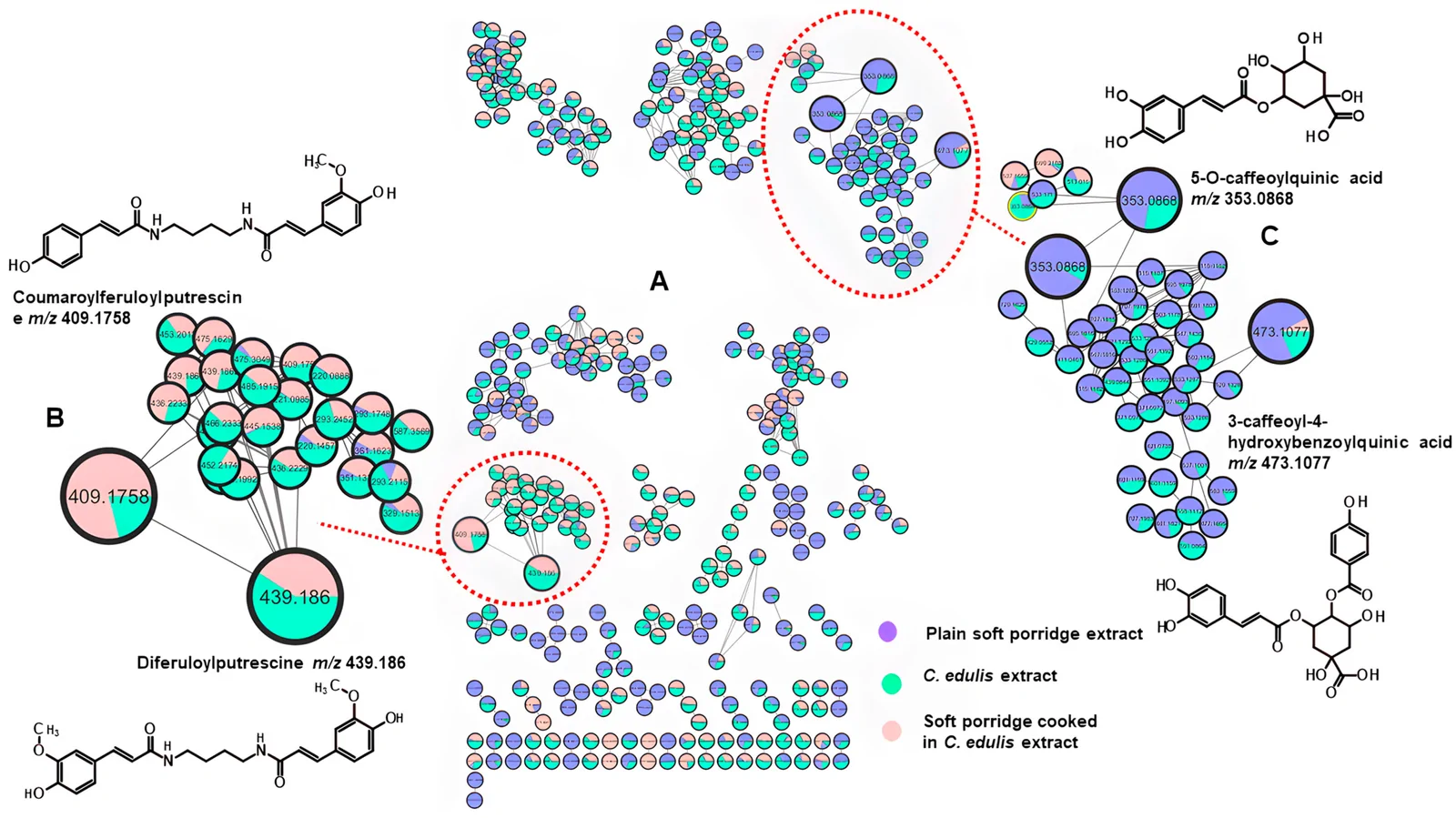
Visual presentation of the metabolic profile of soft porridge prepared in *C.edulis* root extracts (light pink) using the UHPLC-qTOF-MS and feature-based molecular networking. (**A**) Full molecular network and (**B**,**C**) hydroxycinnamoyl derivatives.

**Figure 4:**
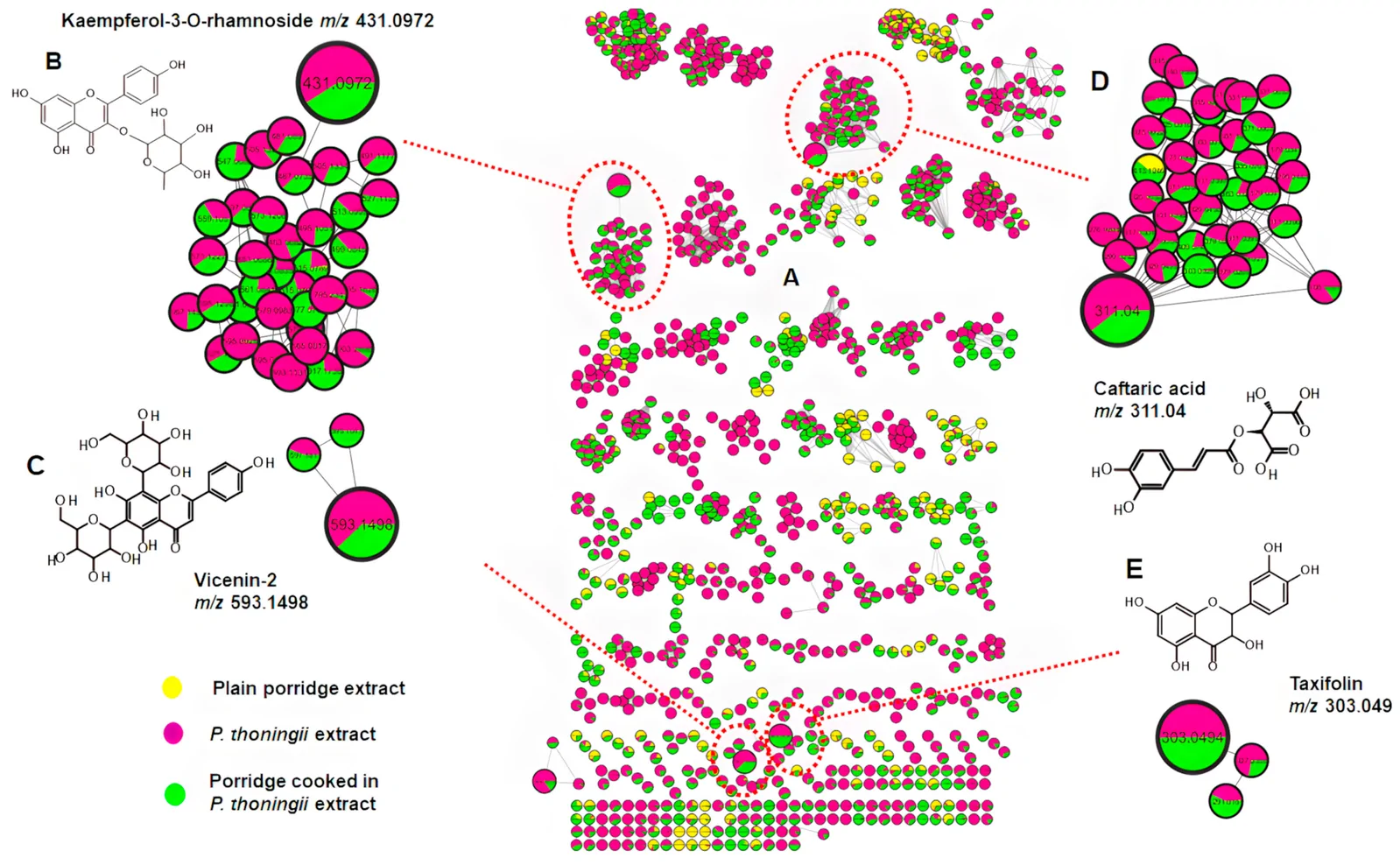
Metabolic profile of soft porridge prepared in *P. thonningii* extract, the corresponding plant extracts and plain soft porridge presented through feature-based molecular networking. (**A**) Full molecular network, (**B**) flavonol, (**C**) C-flavonoid glycoside, (**D**) hydroxycinnamoyl derivatives and (**E**) dihydroflavonol.

Generally, the soft porridge samples prepared in the plant extracts and their corresponding plant materials were found to contain compounds that have been reported to possess health benefits, with most of the compounds putatively belonging to the polyphenols class, as seen in Figure 1, Figure 2, Figure 3 and Figure 4 and Tables S1–S4. Compounds found in more than one plant were only characterised once, although they were also listed in subsequent tables. Most of the compounds detected in the plant extracts were also putatively identified in the corresponding soft porridge, indicating that these metabolites remained detectable after cooking. Only a few compounds (enlarged nodes) representing identified classes were displayed using molecular networking for each plant. All compounds reported in this study were annotated at MSI level 2 confidence according to the Metabolomics Standards Initiative guidelines [27]. The chromatograms for soft porridge prepared in these plants, the corresponding plant extracts and plain soft porridge are shown in Figure S1.

- Characterisation of compounds in soft porridge samples prepared in the root extracts of *A. senegalensis* through the UHPLC-qTOF-MS and feature-based molecular networking

UHPLC-qTOF-MS metabolic profiling of the methanolic extracts of soft porridge samples prepared from *A. senegalensis* root extracts and the corresponding plant extracts revealed a metabolome predominantly consisting of polyphenolic compounds (Table S1; Figure 1). For this plant, plant powder was also added to cooked plain porridge, as reports from surveys [4] suggest that this is one of the methods used to prepare infant food with this plant, and the results are also displayed in Figure 1. However, since this particular sample displayed similar results to soft porridge cooked in plant extracts, only the latter was included in Table S1. It is notable that most compounds observed in the plant methanolic extracts are also present in the corresponding soft porridge samples (as evidenced by the occurrence of green and pink colours in the nodes in Figure 1). Several compounds under the flavan-3-ol subclass of flavonoids (Figure 1B) were putatively identified based on accurate mass and diagnostic fragmentation patterns. A compound at *m*/*z* 577.1337 [M-H]^−^ was tentatively characterised as procyanidin B2 due to fragment ions at *m*/*z* 451 and 289, corresponding to C-ring cleavage and the formation of a deprotonated epicatechin unit. A related monomer at *m*/*z* 289.0706 [M-H]^−^, yielding fragment ions at *m*/*z* 151 and 137 consistent with flavanol C-ring cleavage, was tentatively identified as epicatechin (Figure 1C) [20,28]. Higher oligomers were also detected, including a proanthocyanidin trimer at *m*/*z* 849.2031 [M-H]^−^, characterised by the diagnostic *m*/*z* 289 fragment, and gambiriin C at *m*/*z* 561.1384 [M-H]^−^, supported by fragmentation consistent with interflavanoid bond cleavage [29,30].

Several flavonoid glycosides, as shown in Figure 1D–F, were also tentatively identified, including vitexin-2″-*O*-rhamnoside (*m*/*z* 577.1555 [M-H]^−^), which showed fragment ions at *m*/*z* 413, 293 and 269, indicative of rhamnose loss and the apigenin aglycone, respectively [28]. Orientin-2″-rhamnoside (*m*/*z* 593.1495 [M-H]^−^) displayed sequential sugar losses consistent with a C-glycosyl luteolin derivative. A fragment ion observed at *m*/*z* 473 for this compound suggested consecutive losses of rhamnose (146 Da, *m*/*z* 447) followed by glucose (162 Da, *m*/*z* 289 luteolin aglycone), with ^0,2^A^−^ cross-ring cleavage [31]. Isorhamnetin-3-*O*-glucoside (Figure 1E) (*m*/*z* 477.1026 [M-H]^−^) was putatively characterised by fragment ions at *m*/*z* 314 and 299 typical of methoxylated flavonoid aglycones [32], while myricetin-3-*O*-glucoside (*m*/*z* 479.0818 [M-H]^−^) (Figure 1F) yielded a characteristic aglycone fragment at *m*/*z* 316 following glucose loss (162 Da) [33].

Hydroxycinnamoyl amides (Figure 1G) were also detected, including feruloyltyramine (*m*/*z* 312.1233 [M-H]^−^), showing fragment ions at *m*/*z* 178 and 135 consistent with feruloyl moiety cleavage [34] and *p*-coumaroyltyramine (*m*/*z* 282.1122 [M-H]^−^), tentatively characterised by a diagnostic fragment at *m*/*z* 119 representing dehydrated coumaric acid [35]. Furthermore, a phenolic acid derivative at *m*/*z* 359.097 [M-H]^−^ was tentatively identified as glucosyringic acid, supported by the aglycone fragment at *m*/*z* 197 after glucose loss (162 Da) [33].

### 3.2. Characterisation of Compounds in Soft Porridge Prepared in B. galpinii Leaf Extracts Through the UHPLC-qTOF-MS and Molecular Networking

Compounds putatively identified in *B. galpinii* are presented in Table S2 and Figure 2. UHPLC-qTOF-MS analysis revealed a metabolite profile dominated by different subclasses of flavonoids, including flavanones, flavonols, flavones and flavan-3-ols, phenolic acids and related glycosides. However, most of these compounds were detected only in plant extracts and not in the corresponding porridge samples, as shown in Table S2. Even though some of the compounds were not detected in soft porridge after cooking, reporting the compounds found in the plant extracts is important, as they can pose health concerns for infants fed food containing these plants, depending on the dosage used.

#### 3.2.1. Flavanones and Flavone Glycosides

A compound at *m*/*z* 449.1083 [M-H]^−^ was putatively identified as helicioside A based on its characteristic fragmentation pattern. The primary fragment at *m*/*z* 287 corresponded to the deprotonated 3,5,7,4′-tetrahydroxyflavanone aglycone, while sequential losses of H_2_O (18 Da) and CO (28 Da) generated ions at *m*/*z* 269 and 259, respectively [36]. The ion at *m*/*z* 125 likely resulted from further aglycone cleavage, consistent with reported flavonoid glycoside fragmentation pathways [37]. Similarly, naringenin-7-*O*-glucoside (*m*/*z* 433.1128 [M-H]^−^), as shown in Figure 1B, was putatively identified by its diagnostic aglycone fragment at *m*/*z* 271 following neutral loss of glucose (162 Da) [32]. Apigenin (*m*/*z* 269.0444 [M-H]^−^) displayed a fragment at *m*/*z* 117 attributed to retro-Diels–Alder (RDA) cleavage of the flavone backbone [38].

C- and *O*-glycosylated flavones were also putatively identified in *B. galpinii* and the respective soft porridge samples. Hemiphloin (*m*/*z* 433.113 [M-H]^−^) produced fragments at *m*/*z* 313 (aglycone after glycoside loss) and *m*/*z* 151 (B-ring fragment) [39]. A compound putatively identified as vicenin-2 (*m*/*z* 593.1501 [M-H]^−^) exhibited characteristic cross-ring cleavage fragments at *m*/*z* 473 and 383, typical of di-C-glycosides [40].

#### 3.2.2. Flavonols and Their Glycosides

Several compounds under the flavonols subclass were also putatively identified based on neutral sugar losses and diagnostic aglycone fragments. Compounds tentatively identified as myricetin-3-*O*-arabinoside (*m*/*z* 449.0717 [M-H]^−^) and myricetin-3-*O*-glucoside (*m*/*z* 479.0817 [M-H]^−^) both showed the characteristic aglycone fragment ion at *m*/*z* 316 after the loss of the sugar moieties [33]. Kaempferol-3-*O*-glucoside (*m*/*z* 447.0921 [M-H]^−^), as shown on Figure 1C, showed fragment ions at *m*/*z* 285 and 284 corresponding to the kaempferol aglycone and radical ion [32], while kaempferol-3-*O*-arabinopyranoside (*m*/*z* 417.0811 [M-H]^−^) exhibited *m*/*z* 285 after neutral loss of arabinose (132 Da). Quercetin glycosides were also putatively identified. Quercetin-3-*O*-glucoside (*m*/*z* 463.0875 [M-H]^−^), as shown in Figure 1C, and quercetin-3-*O*-arabinoside (*m*/*z* 433.0769 [M-H]^−^) both generated diagnostic fragments at *m*/*z* 301 and 300 corresponding to the deprotonated aglycone and its radical form [39]. The aglycone quercetin (*m*/*z* 301.0337 [M-H]^−^) displayed an RDA fragment ion at *m*/*z* 151 [41], while myricetin (*m*/*z* 317.0292 [M-H]^−^) was also tentatively characterised by its typical RDA fragments at *m*/*z* 151, 137, and 109 [33]. Dihydromyricetin (*m*/*z* 319.0444 [M-H]^−^) produced RDA fragments at *m*/*z* 193 and 125 as previously reported [42].

#### 3.2.3. Flavan-3-ols and Proanthocyanidins

A compound tentatively identified as epigallocatechin, as observed at *m*/*z* 305.0655 [M-H]^−^ (Figure 1D), showed fragment ions at *m*/*z* 137 and *m*/*z* 125, corresponding to the cleavage of the flavan-3-ol ring [33]. Epicatechin (*m*/*z* 289.071 [M-H]^−^) and procyanidin B2 (*m*/*z* 577.1339 [M-H]^−^) were identified based on characteristic flavan-3-ol fragmentation patterns as previously discussed in Section 3.1.

#### 3.2.4. Phenolic Acids and Related Derivatives

4-caffeoylquinic acid (*m*/*z* 353.0863 [M-H]^−^) was putatively identified based on characteristic fragment ions at *m*/*z* 173 (dehydrated quinic acid), 179 (caffeic acid), and 135 (decarboxylated caffeic acid), consistent with established LC-ESI-MS/MS fragmentation behaviour [43]. Tentatively identified cinnamoyl glycosides, *trans*-*p*-coumaric acid-4-glucoside (*m*/*z* 325.0916 [M-H]^−^), as shown in Figure 1E, showed a fragment at *m*/*z* 119 representative of decarboxylated coumaric acid [44], while, sinapoyl glucose (*m*/*z* 385.1129 [M-H]^−^), as shown in Figure 1F, exhibited a fragment at *m*/*z* 223 corresponding to the sinapic acid moeity after glucose loss [45]. Another phenolic acid glycoside, 12-hydroxyjasmonic acid glucoside (*m*/*z* 387.165 [M-H]^−^ was characterised by a fragment at *m*/*z* 207 corresponding to a dehydrated jasmonate ion after neutral sugar loss [46].

### 3.3. Characterisation of Compounds in Soft Porridge Prepared in C. edulis Root Extracts Through the UHPLC-qTOF-MS and Molecular Networking

In soft porridge prepared with *C. edulis* root extracts and the corresponding plant root extracts, the UHPLC-qTOF-MS profiling enabled putative annotation of several phenolic amides, flavonoid glycosides, chlorogenic acids and lipid-related metabolites (Table S3 and Figure 3). Table S3 and Figure 3 show that most of the compounds identified in the plant extracts and the corresponding soft porridge were also observed in plain maize meal porridge, indicating that most plant-based samples share common compounds. A precursor ion at *m*/*z* 439.1862 [M-H]^−^ was putatively identified as diferuloylputrescine, a hydroxycinnamic acid amide, as shown in Figure 3B. The diagnostic fragment ions at *m*/*z* 149 and 135 correspond to ferulic acid-derived moieties [47]. Another hydroxycinnamoyl amide at *m*/*z* 409.1759 with diagnostic fragments at *m*/*z* 119 (decarboxylated coumaric acid) and 134 (decarboxylated ferulic acid) was tentatively identified as coumaroylferuloylputrescine, as shown in Figure 3B. A related compound at *m*/*z* 436.2229 [M-H]^−^ was tentatively annotated as dicoumaroylspermidine, producing the diagnostic coumaroyl fragment at *m*/*z* 119.

Quercetin-3-*O*-glucoside and quercetin-3-*O*-arabinoside, detected at *m*/*z* 463.0869 and 433.076 [M-H]^−^, respectively, were characterised in Section 3.2. Another quercetin glycoside observed at *m*/*z* 447.0914 [M-H]^−^ was putatively identified as quercetin-3-*O*-rhamnoside. Its MS/MS fragmentation pattern exhibited the same characteristic aglycone fragment ion at *m*/*z* 301, consistent with the other quercetin glycosides already discussed, thereby confirming the presence of the quercetin core structure. A compound at *m*/*z* 431.0968 [M-H]^−^ was putatively identified as vitexin (apigenin-8-C-glucoside). A prominent fragment ion at *m*/*z* 311 from this compound resulted from cross-ring cleavage (^0,3^X^−^) of the C-linked glucose moiety, which distinguishes flavone C-glycosides from O-glycosides [28].

Several chlorogenic acids were also tentatively identified, including a compound at *m*/*z* 353.0868 [M-H]^−^, as shown in Figure 3C, putatively identified as 5-*O*-caffeoylquinic acid. This compound exhibited the typical fragment at *m*/*z* 191 corresponding to deprotonated quinic acid after loss of a caffeoyl unit [44]. A related compound at *m*/*z* 515.1182 [M-H]^−^ was assigned as 4,5-dicaffeoylquinic acid, producing fragments at *m*/*z* 179 (caffeic acid) and 173 (dehydrated quinic acid). Similarly, *m*/*z* 529.1328 [M-H]^−^ was identified as 3-caffeoyl-4-feruloylquinic acid, confirmed by fragment ions at *m*/*z* 193 (ferulic acid) and 173 (dehydrated quinic acid) [44]. A compound at *m*/*z* 473.1077 [M-H]^−^, as shown in Figure 3C, was characterised as 3-caffeoyl-4-hydroxybenzoylquinic acid, supported by fragments at *m*/*z* 137 (hydroxybenzoic acid moiety) and 173 (dehydrated quinic acid) [19]. A lysophospholipid at *m*/*z* 571.287 [M-H]^−^ was annotated as LysoPI (16:0/0:0). The fragment at *m*/*z* 255 corresponds to the palmitoyl (C16:0) acyl chain, confirming its lysoglycerophospholipid structure [48]. A compound at *m*/*z* 539.1396 [M-H]^−^ was putatively identified as di-*O*-syringoyl glucopyranose, producing fragments at *m*/*z* 197 and 183 corresponding to syringic acid and its demethylated derivative, respectively [49].

### 3.4. Characterisation of Compounds in Soft Porridge Prepared in P. thonningii Leaf Extracts Through the UHPLC-qTOF-MS and Molecular Networking

Metabolomic profiling of soft porridge prepared in *P. thonningii* extracts and the plant extract showed a chemically diverse composition dominated by polyphenolic constituents. The metabolite profile was particularly rich in flavonoids (flavonols, flavanones, flavan-3-ols), hydroxycinnamic acid derivatives, hydroxybenzoic acids, lignans, and other secondary metabolites (Table S4). The abundance of compounds from these classes shows that this plant is rich in polyphenols. Figure 4 shows some of the compounds that were annotated in soft porridge samples prepared in *P. thonningii* extracts. Most of the compounds observed in plant extracts were also putatively identified in the corresponding soft porridge. Among the flavonols, several quercetin derivatives, including quercetin-3-*O*-glucoside (*m*/*z* 463.0864 [M-H]^−^) and quercetin-3-*O*-rhamnoside (*m*/*z* 447.0913 [M-H]^−^), were tentatively identified based on characteristic MS/MS fragmentation patterns as already discussed in Section 3.2. Quercetin dihexose was observed at *m*/*z* 625.1396 [M-H]^−^ and showed sequential losses of hexose units to yield fragment ions at *m*/*z* 463 and 301, further confirming the quercetin backbone [32]. Another quercetin derivative, quercetin-3-(6″-malonyl-glucoside), detected at *m*/*z* 549.0869 [M-H]^−^, exhibited a characteristic neutral loss of 86 Da corresponding to a malonyl group, forming *m*/*z* 463 and, subsequently, *m*/*z* 301. Quercetin-3-*O*-acetyldiglucopyranoside at *m*/*z* 667.1496 [M-H]^−^ fragmented to *m*/*z* 505 after hexose loss and showed *m*/*z* 301, confirming the presence of a quercetin aglycone [50]. Isorhamnetin, putatively identified at *m*/*z* 315.0497 [M-H]^−^, produced fragments at *m*/*z* 300 and 271, due to demethylation and CO_2_ loss, respectively [51]. Isorhamnetin-3-*O*-glucoside at *m*/*z* 477.1026 [M-H]^−^ showed an aglycone fragment at *m*/*z* 314 following hexose cleavage [52], while Isorhamnetin-3,4-diglucoside at *m*/*z* 639.1547 [M-H]^−^ showed sequential hexose losses to form fragment ions at *m*/*z* 477 and 315. Kaempferol derivatives tentatively identified at *m*/*z* 431.0968 [M-H]^−^ as shown in Figure 4B and *m*/*z* 417.0811 [M-H]^−^, corresponding to kaempferol-3-*O*-rhamnoside and kaempferol-3-*O*-arabinopyranoside, respectively, both producing characteristic aglycone fragments at *m*/*z* 284 and 285 as detailed in Section 3.2.

Naringenin-7-O-glucoside tentatively detected at *m*/*z* 433.1127 [M-H]^−^ and vicenin-2 (Figure 4C) at *m*/*z* 593.1501 [M-H]^−^ have been characterised in Section 3.2. Flavan-3-ols and proanthocyanidins were also predominant in the extracts containing *P. thonningii*, with epicatechin at *m*/*z* 289.0706 [M-H]^−^ and procyanidin B (*m*/*z* 577.1337 [M-H]^−^) having already been characterised in Section 3.1 and Section 3.2. Another dimeric proanthocyanidin, putatively identified as procyanidin A1 at *m*/*z* 579.1490 [M-H]^−^, yielded the diagnostic monomeric fragment at *m*/*z* 289 via interflavan bond cleavage [28]. On the other hand, a compound tentatively characterised as epicatechin–epiafzelechin dimer at *m*/*z* 561.1382 [M-H]^−^ showed a peak at *m*/*z* 289, which is an RDA cleavage product.

Hydroxycinnamic acid derivatives putatively annotated in *P. thonningii* extracts, which have been identified in the other plants characterised in this study (Section 3.2 and Section 3.3), include *trans-p*-coumaric acid-4-glucoside at *m*/*z* 325.0916 [M-H]^−^ and sinapoyl glucose at *m*/*z* 385.1129 [M-H]^−^. Caftaric acid (*m*/*z* 311.0396 [M-H]^−^), as shown in Figure 4D, and coutaric acid (*m*/*z* 295.0445) showed fragment ions at *m*/*z* 135 and *m*/*z* 119, respectively, which are indicative of the presence of caffeoyl and coumaroyl moieties, respectively [53]. Tentatively identified hydroxybenzoic acid derivatives included syringic acid-O-glucoside at *m*/*z* 359.0972 [M-H]^−^ and gentisic acid glucoside at *m*/*z* 315.0710 [M-H]^−^, producing fragments at *m*/*z* 152 and 108. Galloyl glucose at *m*/*z* 331.0660 was putatively identified with the fragment ion at *m*/*z* 125, corresponding to decarboxylated gallic acid [49].

**Figure 5:**
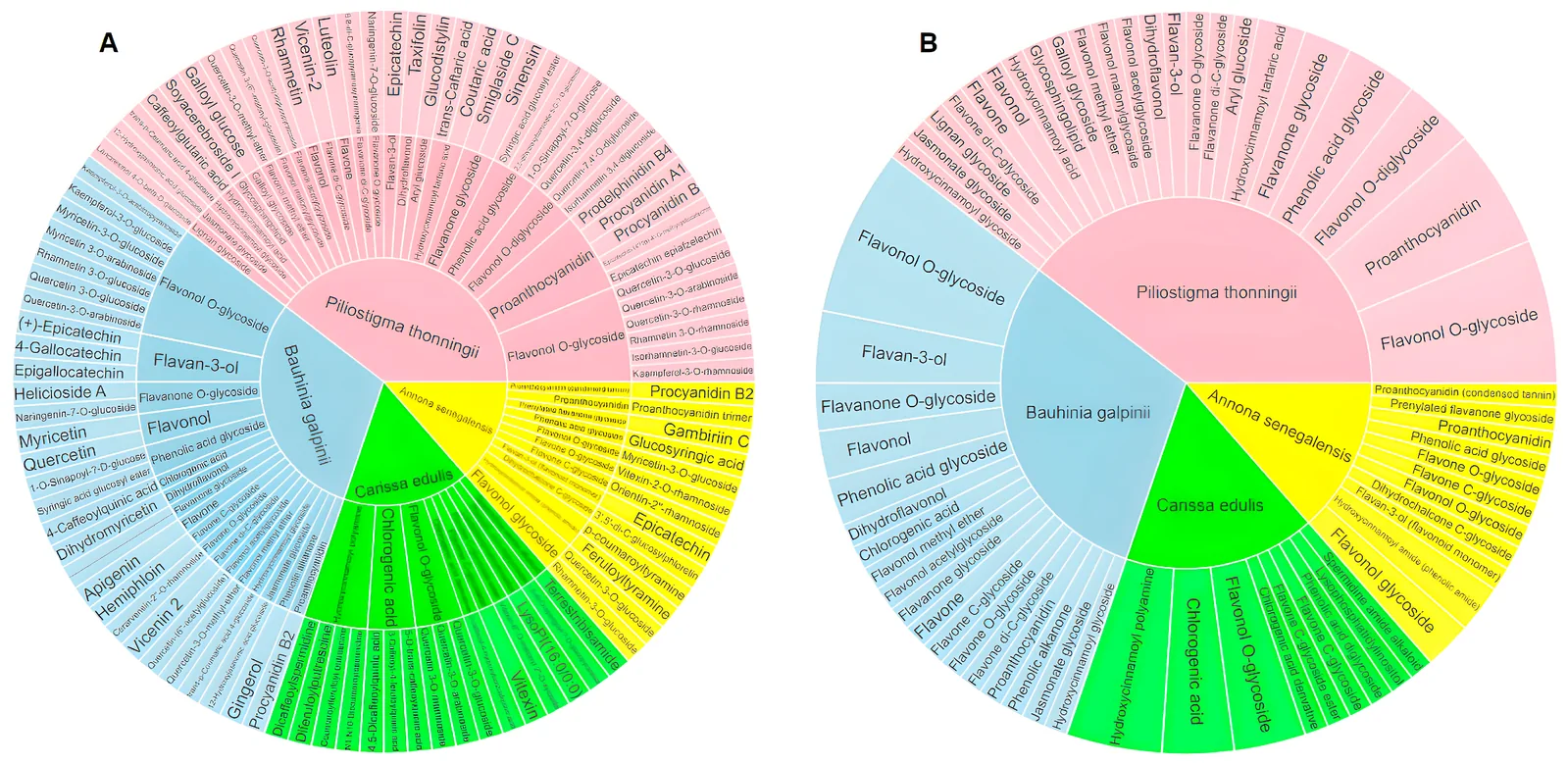
A sunburst plot summarising secondary metabolites putatively identified from *B. galpinii*, *A. senegalensis*, *C. edulis*, and *P. thonningi* and the corresponding soft porridge samples, with (**A**) showing the plant-chemical class-compound hierarchy, (**B**) plant-chemical class hierarchy.

Dihydroflavonols were also detected, including taxifolin at *m*/*z* 303.0493 [M-H]^−^ as shown in Figure 4E, and its glycoside glucodistylin at *m*/*z* 465.1020 [M-H]^−^. Both compounds exhibited typical characteristic fragment ions at *m*/*z* 285 and 125 [54]. Figure 5 shows a summary of the different polyphenol subclasses of the compounds putatively identified in this study.

### 3.5. Cytotoxic Effects of Aqueous Root Extract of A. senegalensis and Aqueous Leaves Extract of P. thonningii

**Figure 6:**
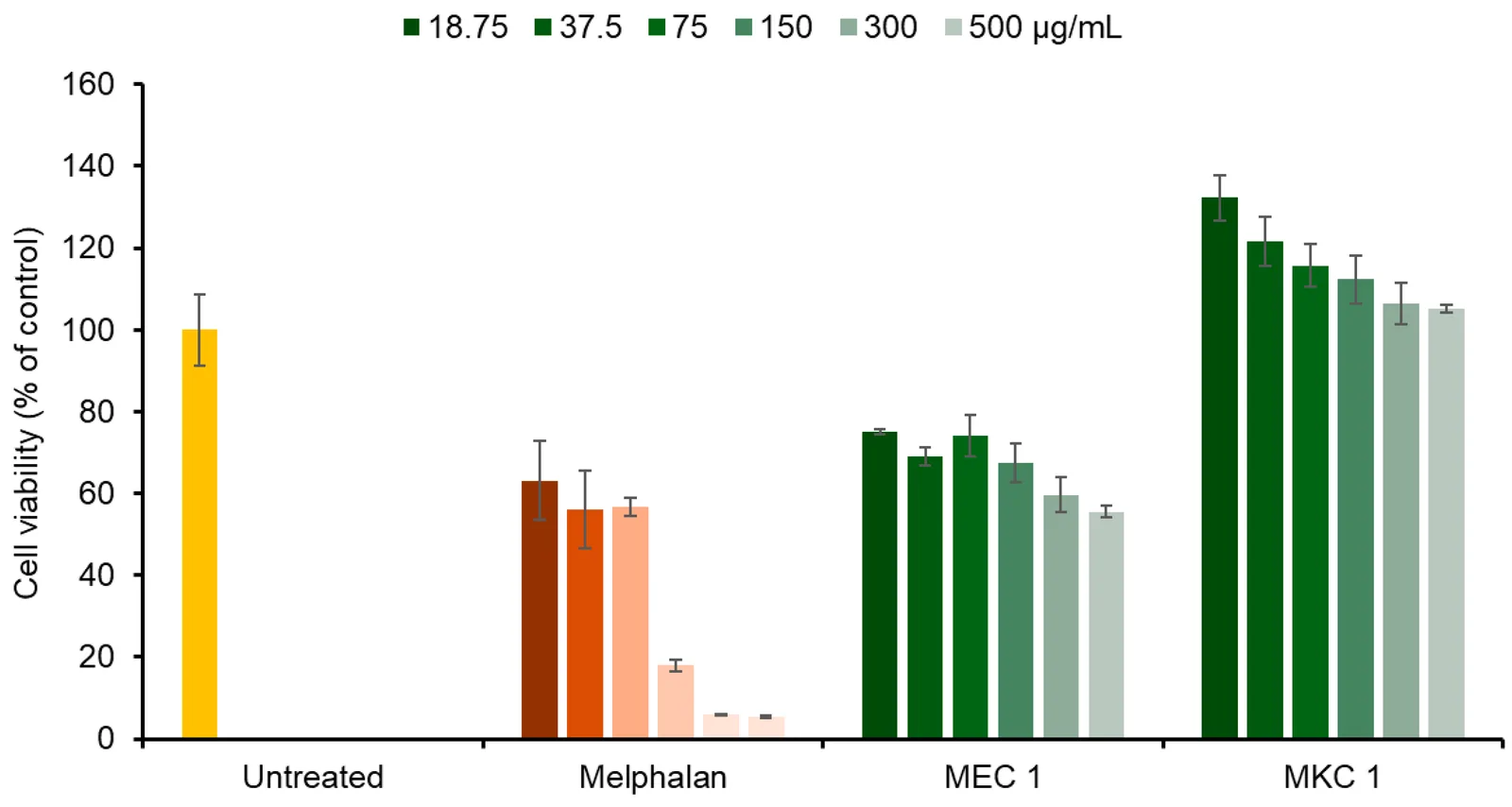
Cytotoxicity of plant extract against Vero cells after 48 h of treatment. *Annona senegalensis*-MEC1, *Piliostigma thonningii*-MKC1.

Although phytochemical profiling of the investigated plants and their corresponding soft porridge formulations did not reveal the presence of known toxic compounds such as some alkaloids and saponins, chemical characterisation alone is insufficient to confirm biological safety. Therefore, preliminary cytotoxicity was assessed for two selected plants, namely, *A. senegalensis* and *P. thonningii*, to evaluate the potential toxicity of their aqueous extracts. These two plants were selected because they were observed to have most of the putatively identified compounds persisting after cooking, suggesting that any potentially toxic molecules may also remain in the prepared soft porridges. Furthermore, a recent ethnobotanical survey by Shikwambana et al. [4] reported that *A. senegalensis* and *P. thonningii* were among the more frequently used in traditional infant healthcare practices, highlighting their greater ethnomedicinal relevance and making them priority candidates for safety evaluation. The MTT assay revealed unique cytotoxic effects of aqueous root extract of *A. senegalensis* (MEC1) and aqueous leaves extract of *P. thonningii* (MKC1) compared to the negative and the positive controls against a concentration range of 18.75–500 µg mL^−1^ after 48 h (Figure 6). The negative control (untreated cells) maintained 100% cell viability throughout the experiment. The positive control (melphalan) demonstrated potent, concentration-dependent cell death, with an IC_50_ of 87. 8366 µg mL^−1^. Moreover, cell viability declined gradually from 63.09% at 18.75 µg mL^−1^ to 5.43% at 500 µg mL^−1^.

MEC1 exhibited a concentration-dependent decrease in cell viability, from 75.13% at 18.75 µg mL^−1^ to 55.57% at 500 µg mL^−1^. As cell viability remained above 50% at all tested concentrations, the IC_50_ was estimated to be >500 µg mL^−1^. As a result, MEC1 was considered inactive, with approximately 4 times lower potency than the positive control. MKC1 also exhibited no cytotoxicity, and cell viability was higher than in the untreated control. Cell viability at 18.75 µg mL^−1^ was 132.33%, and at 500 µg mL^−1^ was 105.21% (Figure 6). Melphalan and MEC1 showed concentration-dependent numerical reductions in cell viability, but these were not statistically significant (*p* > 0.05) and MKC 1 showed no evidence of cytotoxicity at the concentrations tested.

## 4. Discussion

The metabolomic profiling of soft porridge samples prepared in plant extracts traditionally used in infant foods revealed a complex chemical composition dominated by polyphenolic compounds. Across all investigated plants, flavonoids, phenolic acids and hydroxycinnamic acid derivatives were consistently detected, reflecting their widespread occurrence due to shared biosynthetic pathways in plants [55]. While many of these compounds have been widely reported to possess antioxidant [56], anti-inflammatory [57] and cytoprotective properties [58], identifying only beneficial compounds does not eliminate the possibility of toxic constituents. Moreover, despite the well-known health benefits of these compounds in adults, such bioactivity does not necessarily translate into safety in infants. Polyphenols, particularly condensed tannins, are known to interact with proteins and digestive enzymes, potentially influencing nutrient absorption [59]. Infants have immature hepatic metabolism, renal clearance, and gut microbiota composition, which may alter the biotransformation and systemic exposure of these compounds. Currently, there is a lack of direct toxicological or dose–response data for these metabolites in neonatal populations. Moreover, while the metabolomic profile suggests the presence of bioactive polyphenols retained after cooking, the safety of these preparations for infant consumption cannot be conclusively established without targeted toxicological and pharmacokinetic studies.

Notably, most metabolites detected in *B. galpini* plant extracts were not detected in the corresponding soft porridge samples. This may suggest that some compounds were partially degraded during cooking, or that their concentrations were very low in the prepared soft porridge. Moreover, the food matrix has been reported to influence the retention and extractability of phytochemicals through interactions with food components such as proteins, starch, and dietary fibre [60,61]. In contrast, the persistence of many metabolites in the other soft porridge samples indicates that these compounds remained detectable after cooking. However, the extent to which interactions with the food matrix contributed to their retention or influenced their biological activity was not investigated in the present study and needs further investigation. As interactions between phytochemicals and the food matrix may also influence their bioaccessibility [62], future studies are needed to determine how these interactions affect the exposure and potential biological effects of bioactive compounds in infants consuming these traditional preparations.

In untargeted metabolomics, compound identification depends on the availability of high-quality reference spectra and well-curated databases, therefore, some potentially toxic metabolites might have remained unannotated. This limitation underscores the need for further structural elucidation studies to comprehensively assess the safety of infant food prepared from plant extracts. Moreover, compounds with known toxicological relevance, including certain alkaloids [63] and saponins, may have remained unidentified because the analysis in this study was performed in negative ionisation mode, whereas these compound classes generally exhibit improved ionisation in positive mode [64]. Notably, many putatively identified compounds persisted after cooking, suggesting that infants consuming such preparations may be exposed to a variety of bioactive phytochemicals derived from medicinal plants. However, the safety of such molecules will depend on their concentrations in infant food and yet-to-be-established dose–response data for neonatal populations. Factors such as dosage, bioavailability, and long-term exposure, therefore, remain critical considerations, particularly in the context of repeated dietary intake. In the absence of rigorous phytochemical screening and comprehensive neonatal safety evaluations, incorporating such plant extracts into infant foods should be approached with caution.

Although phytochemical profiling did not reveal the presence of known toxic compounds, chemical characterisation alone was insufficient to confirm biological safety. Cytotoxicity assessment provided additional preliminary insight into the potential biological effects of selected plant extracts on Vero cells. Vero cells are commonly used and a sensitive mammalian screening model, but they are of non-human primate origin. Therefore, the interpretation of the cytotoxicity results should be considered an initial indication of general mammalian toxicity, not a direct predictor of human risk. Furthermore, the concentrations evaluated in this study (18.75–500 µg mL^−1^) were not intended to represent actual doses ingested by infants. Quantification of exposure was challenging because traditional remedies are prepared using non-standardised measures such as “a handful of leaves” or “2–4 roots,” leading to significant variation in extract concentrations [4,9]. Instead, they were chosen to identify concentration-dependent cytotoxic effects and determine IC_50_ values across a range spanning low to relatively high extract concentrations reported in similar ethnopharmacological cytotoxicity studies. The aqueous root extract of *A. senegalensis* (MEC1) exhibited a concentration-dependent decrease in cell viability, with an IC_50_ greater than 500 µg mL^−1^. The observed reduction in cell viability may be associated with polar phytochemicals such as tannins, flavonoids and phenolic acids (Tables S1–S4) that are known to induce mild oxidative stress or mitochondrial disruption [65].

Despite the observed (IC_50_ > 500 µg mL^−1^), evidence from rodent models indicates that root bark extracts of *A. senegalensis* may exhibit subacute hepatotoxicity at higher oral doses, potentially due to compounds such as diterpenoids and saponins that are poorly cleared by neonatal metabolic pathways [66]. Furthermore, subacute toxicity studies have demonstrated that doses of 400 mg kg^−1^ and above had histopathological signs of hepatocellular degeneration and necrosis, whereas lower doses (50–100 mg kg^−1^) were mostly without overt liver injury [66]. The observed relatively low cytotoxicity in Vero cells is consistent with rodent data indicating safety at lower doses, although toxicity may be observed at higher doses or supra-therapeutic exposures. These findings raise concerns regarding cumulative exposure and potential adverse effects in infants, particularly in undernourished populations where pharmacokinetic safeguards may be compromised. Therefore, caution is warranted when considering the use of such extracts in infant food preparations.

In contrast, the aqueous extract of *P. thonningii* (MKC1) showed no cytotoxicity and demonstrated increased cell viability relative to untreated controls, suggesting potential cytoprotective or metabolic-stimulating effects. Furthermore, higher cell viability in MKC1 could be attributed to the plant’s high polyphenolic content (Table S4). However, it should be noted that plant extracts and their compounds, especially polyphenols, flavonoids, thiols, and other redox-active compounds, can directly reduce MTT or interfere with the assay chemistry. This can result in false-positive increases in absorbance that do not reflect true cell viability or metabolic activity. Furthermore, coloured compounds in crude extracts may absorb at or near the detection wavelength, inflating the apparent viability [67]. Therefore, future studies should utilise orthogonal viability assays, such as sulforhodamine B (SRB), resazurin/alamar blue, adenosine triphosphate (ATP)-based luminescence, lactate dehydrogenase (LDH) release, or live–dead fluorescence imaging, to confirm whether the observed effects reflect true cell viability, metabolic changes, or are assay artefacts. The findings of the current study are similar to previous reports on the water fractions of *P. thonningii*’s stem bark, which showed low lethality (LD_50_ > 5000 mg kg^−1^) by reducing oxidative stress in cells, thereby increasing mitochondrial activity and enhancing cellular resilience [65]. Therefore, the results suggest potential nutraceutical value of soft porridge formulations, although their safety in infants has not yet been established. Future research should focus on expanding toxicological assessment using human neonatal renal or hepatic models that directly capture infant-specific toxicokinetics and developmental susceptibility based on quantitative data of the identified compounds. The solvent used in this study (80% methanol) may have provided a wider coverage of metabolites; however, it would be beneficial to analyse water extracts on the LC-MS for a more direct comparison between the chemical composition and the observed cytotoxicity results. Furthermore, future studies should conduct complementary bioassays, such as the brine shrimp lethality assay and employ sequential extraction techniques to isolate and characterise bioactive and potentially toxic constituents. Such approaches will be essential for identifying cytotoxic components and establishing the safety of traditional food preparations for infant consumption.

## 5. Conclusions

This study profiled the secondary metabolites in soft porridges prepared with extracts from plants commonly added to infant diets in Limpopo, such as *A. senegalensis* roots, *P. thonningii* leaves, *C. edulis* roots and *B. galpinii* leaves to assess their safety for infants. Different classes of polyphenols were identified by UHPLC-qTOF-MS and molecular networking, with several compounds persisting after cooking, particularly in soft porridge of *A. senegalensis* and *P. thonningii*. However, the putative identification of compounds with reported health benefits does not necessarily indicate that they are safe for use in infant diets. The cytotoxicity assay of the aqueous extracts using Vero cells showed no cytotoxicity for *P. thonningii*, whereas *A. senegalensis* extract was inactive (IC_50_ > 500 µg mL^−1^). These findings suggest that *A. senegalensis* should be investigated further for neonatal safety. They also highlight the importance of exercising caution when incorporating medicinal plants into infant foods in the absence of adequate safety data. Overall, this study provided a foundation for future investigations into the chemical composition, biological activity and safety of medicinal plants traditionally used in infant foods.

## Data Availability

The datasets obtained during this study can be made available from the corresponding author upon reasonable request.

## Supplementary Materials

The following supporting information can be downloaded at: https://www.mdpi.com/article/10.3390/metabo16090606/s1, Figure S1: Total ion chromatograms of plant extracts, soft porridge prepared in plant extracts and plain maize porridge samples. (A) *B. galpinii*, (B) *A. senegalensis*, (C) *C. edulis*, (D) *P. thonningii*; Table S1: Putative identification of secondary metabolites in porridge prepared in *A. senegalensis* extract using the UHPLC-qTOF-MS; Table S2: Putative identification of secondary metabolites in porridge prepared in *B. galpinii* extract using the UHPLC-qTOF-MS; Table S3: Putative identification of secondary metabolites in *C. edulis* using the UHPLC-qTOF-MS; Table S4: Putative identification of secondary metabolites in porridge prepared in *P. thonningii* extract using the UHPLC-qTOF-MS; Table S5: Summary of IC_50_ values and viability endpoints on Vero cells.
