# Supplementary material for: Metabolomic Profiling of Medicinal Plant-Based Soft Porridge and Evaluation of Cytotoxicity
Source: Metabolites. 2026 Aug 25;16(9):606. doi: 10.3390/metabo16090606 (PMC13609092; doi:10.3390/metabo16090606)
Supplement: Supplementary file 1 [file metabolites-16-00606-s001.zip › metabolites-4457529-supplementary.pdf]

Supplementary information

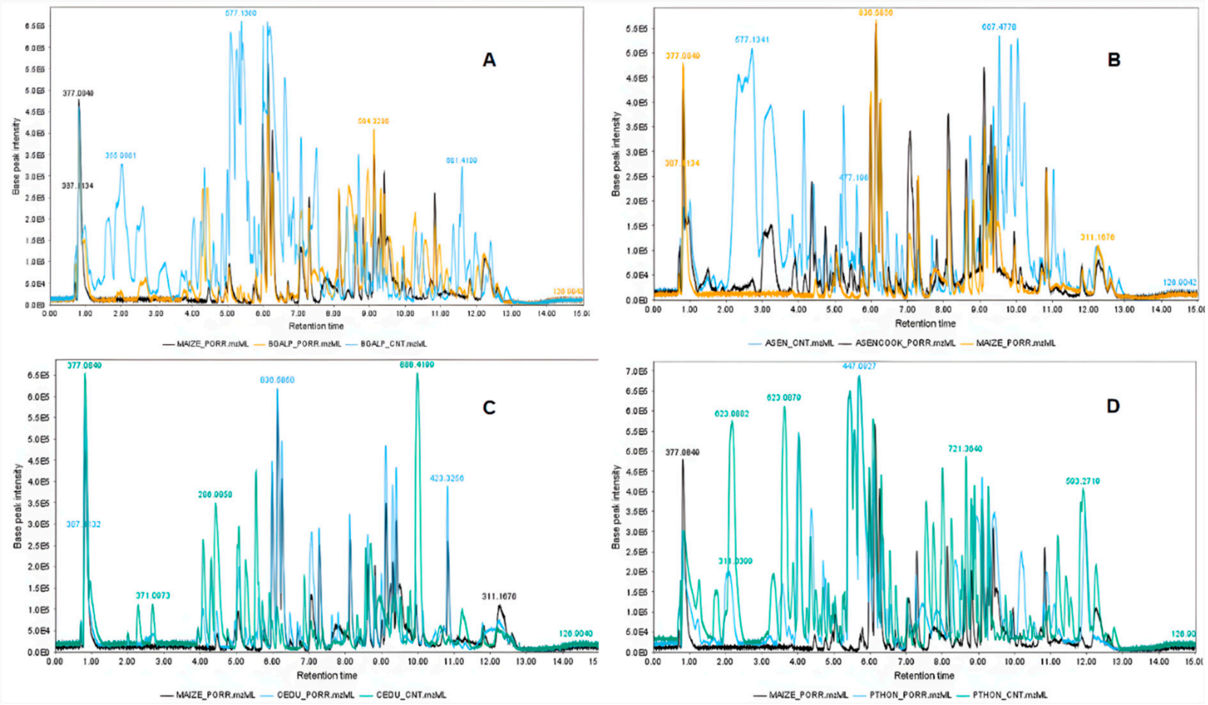

Figure S1. Total ion chromatograms of plant extracts, soft porridge prepared in plant extracts and plain maize soft porridge samples. (A) *B. galpinii*, (B) *A. senegalensis*, (C) *C. edulis*, (D) *P. thonningii*.

GNPS2 feature-based molecular networking task IDs:

B. galpinii: https://gnps2.org/status?task=22454a3a9f3348a492fce6200179ffd6

A.senegalensis: https://gnps2.org/status?task=41621625d60d43d78f5a10086f02dbde

C. edulis: https://gnps2.org/status?task=82e86236b3394b489f242d003bd23a02

P.thonningii: https://gnps2.org/status?task=309f813ed70e4dfba92a2a6b0b9e1309

Table S1. Putative identification of secondary metabolites in porridge prepared in *A. senegalensis* extract using the UHPLC-qTOF-MS.

Experimental mass (*m/z*)	rt (min)	Compound Name	Fragment ions (*m/z*)	Molecular Formula	Theoretical mass	Mass error (ppm)	Plant extract	Soft porridge in plant extract	Plain soft porridge extract
577.1337	4.04	Procyanidin B2	457, 407, 289	$C_{30}H_{26}O_{12}$	577.1346	-3.29	✓	✓	-
289.0706	5.14	Epicatechin	151,137	$C_{15}H_{14}O_6$	289.0712	-2.08	✓	✓	-
312.1233	5.80	Feruloyltyramine	190,178, 135	$C_{18}H_{19}NO_4$	312.1241	-2.56	✓	✓	-
577.1555	5.38	Vitexin-2''-*O*-rhamnoside	413, 293, 269	$C_{27}H_{30}O_{14}$	577.1563	-1.35	✓	✓	✓
477.1026	5.59	Isorhamnetin-3-*O*-glucoside	314, 299	$C_{22}H_{22}O_{12}$	477.1039	-2.62	✓	✓	✓
282.1122	5.70	*p*-coumaroyltyramine	119	$C_{17}H_{17}NO_3$	282.1130	-2.98	✓	✓	✓
597.1812	9.18	3',5'-di-C-glucosylphloretin	387, 357	$C_{27}H_{34}O_{15}$	597.1825	-2.50	✓	✓	✓
463.0864	5.64	Qurcetin-3-*O*-glucoside	331,161	$C_{21}H_{20}O_{12}$	463.0876	-2.59	✓	✓	-
359.0972	3.52	Glucosyringic acid	197	$C_{15}H_{20}O_{10}$	359.0984	-3.82	✓	✓	-
849.2031	4.33	Proanthocyanidin trimer	289	$C_{45}H_{38}O_{17}$	849.2036	-0.73	✓	✓	-
593.1495	5.12	Orientin-2''-rhamnoside	473,357	$C_{27}H_{30}O_{15}$	593.1512	-3.70	✓	✓	-
561.1384	4.76	Gambiriin C	289	$C_{30}H_{26}O_{11}$	561.1402	-3.98	✓	✓	-
479.0818	5.10	Myricetin-3-*O*-glucoside	316	$C_{21}H_{20}O_{13}$	479.0831	-2.33	✓	✓	✓

√- present; - absent

Table S2. Putative identification of secondary metabolites in porridge prepared in *B. galpinii* extract using the UHPLC-qTOF-MS.

Experimental mass (*m/z*)	rt (min)	Compound Name	Fragment ions (*m/z*)	Molecular Formula	Theoretical [M-H]-	Mass error (ppm)	Plant extract	Soft porridge in plant extract	Plain soft porridge extract
449.1083	4.28	Helicioside A	287, 269, 259, 125	$C_{21}H_{22}O_{11}$	449.1089	-1.34	✓	-	-
433.1128	4.95	Naringenin-7-*O*-glucoside	193, 271	$C_{21}H_{22}O_{10}$	433.1140	-2.77	✓	-	-
577.1337	2.37	Procyanidin B2	289, 407, 125, 245	$C_{30}H_{26}O_{12}$	577.1346	-3.29	✓	-	-
269.0444	6.61	Apigenin	117	$C_{15}H_{10}O_{5}$	269.0455	-4.08	✓	✓	-
305.0655	1.51	Epigallocatechin	125, 137, 166, 137,	$C_{15}H_{14}O_{7}$	305.0667	-3.86	✓	-	-
449.0717	5.30	Myricetin-3-*O*-arabinoside	316	$C_{20}H_{18}O_{12}$	449.0726	-1.89	✓	-	-
447.0921	5.66	Kaempferol-3-*O*-glucoside	284, 285	$C_{21}H_{20}O_{11}$	447.0933	-2.65	✓	✓	✓
417.0811	5.81	Kaempferol-3-*O*-arabinopyranoside	285, 255, 209, 227	$C_{20}H_{18}O_{10}$	417.0827	-3.88	✓	✓	-
477.1026	5.60	Isorhamnetin-3-*O*-glucoside	314, 299, 215, 271	$C_{22}H_{22}O_{12}$	477.1039	-2.62	✓	✓	✓
463.0875	5.42	Quercetin-3-*O*-glucoside	300, 301	$C_{21}H_{20}O_{12}$	463.0882	-1.51	✓	✓	✓
433.0769	5.59	Quercetin-3-*O*-arabinoside	300, 301, 271, 255	$C_{20}H_{18}O_{11}$	433.0776	-1.70	✓	✓	✓
353.0863	4.86	4-Caffeoylquinic acid	179,173,135	$C_{16}H_{18}O_{9}$	353.0873	-2.83	✓	✓	-
319.0444	4.21	Dihydromyricetin	193,191,125	$C_{15}H_{12}O_{8}$	319.0459	-2.95	✓	-	-
387.1649	4.72	12-Hydroxyjasmonic acid glucoside	163, 207	$C_{18}H_{28}O_{9}$	387.1661	-2.73	✓	✓	-
305.0656	1.51	Gallocatechin	125,137,165	$C_{15}H_{14}O_{7}$	305.0667	-2.22	✓	-	-

433.1128	5.04	Hemiphloin	313, 217, 151, 193,	$C_{21}H_{22}O_{10}$	433.1140	-2.36	✓	_	_
385.1129	4.76	Sinapoyl glucose	223	$C_{17}H_{22}O_{10}$	385.1140	-2.65	✓	✓	✓
593.1501	1.36	Vicenin-2	383, 473	$C_{27}H_{30}O_{15}$	593.1512	-2.01	✓	-	-
301.0337	6.08	Quercetin	151	$C_{15}H_{10}O_{7}$	301.0349	-3.99	✓	✓	✓
325.0916	4.04	*trans*-*p*-Coumaric acid-4-glucoside	119	$C_{15}H_{18}O_{8}$	325.0928	-2.74	✓	_	_
317.0292	5.67	Myricetin	151, 137, 109	$C_{15}H_{10}O_{8}$	317.0298	-1.89	✓	_	_
479.0817	5.3	Myricetin-3-*O*-glucoside	316	$C_{21}H_{20}O_{13}$	479.0831	-2.33	✓	_	_
289.0706	4.39	(+)-Epicatechin	123,109	$C_{15}H_{14}O_{6}$	289.0712	-2.08	✓	_	✓
547.1448	5.32	Cerarvensin-2''-*O*-rhamnoside	383	$C_{26}H_{28}O_{13}$	547.1457	-1.31	✓	✓	-
359.0968	3.03	Syringic acid glucosyl ester	138,182	$C_{15}H_{20}O_{10}$	359.0984	-3.82	✓	✓	✓
315.0498	6.29	3-*O*-Methylquercetin	271	$C_{16}H_{12}O_{7}$	315.0510	-3.26	✓	✓	✓
505.0979	5.55	Quercetin-(6''-acetylglucoside)	300	$C_{23}H_{22}O_{13}$	505.0988	-1.51	✓	_	_

✓- present; - absent

Table S3. Putative identification of secondary metabolites in *C. edulis* using the UHPLC-qTOF-MS.

**Experimental mass (*m/z*)**	**rt** (min)	**Compound name**	**Fragment ions (*m/z*)**	**Molecular Formula**	**Theoretical [M-H]-**	**Mass error (ppm)**	**Plant extract**	**Soft porridge in plant extract**	**Plain soft porridge extract**
439.1862	6.00	Diferuloylputrescine	149, 135	$C_{24}H_{28}N_2O_6$	439.1875	-2.96	√	√	-
431.0968	5.24	Vitexin	311, 283	$C_{21}H_{20}O_{10}$	431.0984	-3.71	√	√	√
463.0869	5.46	Quercetin-3-*O*-glucoside	300, 301	$C_{21}H_{20}O_{12}$	463.0882	-2.81	√	√	√
571.2866	9.09	LysoPI(16:0/0:0)	255	$C_{25}H_{49}O_{12}P$	571.2889	-4.03	√	√	√
409.1759	4.93	Coumaroylferuloyl putrescine	119,134	$C_{23}H_{26}N2O_5$	409.1769	-2.19	√	√	-
436.2229	5.04	Dicoumaroylspermidine	290,119	$C_{25}H_{31}N3O_4$	436.2242	-2.71	√	√	-
473.1077	5.19	3-caffeoyl-4-hydroxybenzoylquinic acid	137,173	$C_{23}H_{22}O_{11}$	473.1089	-1.98	√	√	√
468.2125	4.66	Dicaffeoylspermidine	306,332,135	$C_{25}H_{31}N_3O_6$	468.2140	-3.20	√	√	-
353.0868	4.08	5-*O*-*trans*-caffeoylquinic acid	191	$C_{16}H_{18}O_9$	353.0873	-1.42	√	-	√
433.0761	5.58	Quercetin-3-*O*-arabinoside	300	$C_{20}H_{18}O_{11}$	433.0776	-3.78	√	√	√
515.1182	5.83	4,5-Dicaffeoylquinic acid	179,173	$C_{25}H_{24}O_{12}$	515.1195	-2.52	√	-	√
539.1396	5.27	di-*O*-syringoylglucopyranose	197,183	$C_{24}H_{28}O_{14}$	539.1406	-1.17	√	-	√
529.1328	5.57	3-Caffeoyl-4-feruloylquinic acid	193,173	$C_{26}H_{26}O_{12}$	529.1352	-4.06	√	-	√
431.0968	5.24	Vitexin	311, 283	$C_{21}H_{20}O_{10}$	431.0984	-3.18	√	√	√
447.0914	5.72	Quercetin-3-*O*-rhamnoside	300, 301	$C_{21}H_{20}O_{11}$	447.0933	-2.87	√	√	√

√- present; - absent

Table S4. Putative identification of secondary metabolites in porridge prepared in *P. thonningii* extract using the UHPLC-qTOF-MS.

**Experimental mass (*m/z*)**	**rt (min)**	**Compound name**	**Fragment ions (*m/z*)**	**Molecular Formula**	**Theoretical [M-H]-**	**Mass error (ppm)**	**Plant extract**	**Soft porridge in plant extract**	**Plain soft porridge extract**
315.0497	6.27	Isorhamnetin	271, 300, 255	$C_{16}H_{12}O_7$	315.0505	-2.54	✓	✓	-
579.1490	4.38	Procyanidin A1	289	$C_{30}H_{28}O_{12}$	579.1508	-3.11	✓	✓	-
577.1337	5.21	Procyanidin B	289, 245, 125	$C_{30}H_{26}O_{12}$	577.1346	-2.60	✓	✓	-
712.5349	11.1	Soyacerebroside I	550, 532	$C_{40}H_{75}NO_9$	712.5369	-2.82	✓	-	-
463.0864	5.83	Quercetin-3-*O*-glucoside	300, 301	$C_{21}H_{20}O_{12}$	463.0882	-3.89	✓	✓	-
433.1127	5.06	Naringeni-7-*O*-glucoside	271,151	$C_{21}H_{22}O_{10}$	433.1140	-3.05	✓	✓	-
819.2335	6.18	Smiglaside C	777, 759,175	$C_{38}H_{44}O_{20}$	819.2353	-2.22	✓	-	✓
607.1446	3.85	Epicatechin-(4beta->8)-4'-O-methylgallocatechin	125,287,161	$C_{31}H_{28}O_{13}$	607.1457	-2.82	✓	✓	-
387.1646	4.71	12-Hydroxyjasmonic acid glucoside	163, 207	$C_{18}H_{28}O_9$	387.1661	-2.73	✓	✓	-
289.0706	4.38	Epicatechin	123,125,109	$C_{15}H_{14}O_6$	289.0712	-2.63	✓	✓	-
595.1647	4.42	6,8-di-C-glucopyranosyl naringenin	355	$C_{27}H_{32}O_{15}$	595.1668	-3.10	✓	✓	-
285.0389	6.19	Luteolin	133	$C_{15}H_{10}O_6$	285.0399	-3.51	✓	✓	-
385.1129	4.76	1-*O*-Sinapoyl glucose	208,164	$C_{17}H_{22}O_{10}$	385.1140	-2.65	✓	✓	✓
465.1020	4.08	Glucodistylin	285, 125	$C_{21}H_{22}O_{12}$	465.1039	-3.98	✓	✓	-
593.1501	4.79	Vicenin-2	385, 353	$C_{27}H_{30}O_{15}$	593.1512	-2.01	✓	✓	-
577.1327	2.37	Procyanidin B2	289, 407, 125, 245	$C_{30}H_{26}O_{12}$	577.1346	-3.29	✓	✓	-
303.0493	5.04	Taxifolin	285, 125	$C_{15}H_{12}O_7$	303.0505	-3.96	✓	✓	-
449.1082	4.27	Sinensin	259,173	$C_{21}H_{22}O_{11}$	449.1089	-2.08	✓	✓	-
431.0968	5.99	Kaempferol-3-*O*-rhamnoside	284, 255	$C_{21}H_{20}O_{10}$	431.0984	-3.18	✓	✓	-

417.0811	5.76	Kaempferol-3-*O*-arabinopyranoside	285, 284, 255	$C_{20}H_{18}O_{10}$	417.0822	-2.64	✓	✓	-
625.1396	5.08	Quercetin dihexose	463, 300, 301	$C_{27}H_{30}O_{17}$	625.1410	-1.64	✓	✓	-
505.0973	5.52	Quercetin-3-*O*-acetylglucoside	300	$C_{23}H_{22}O_{13}$	505.0982	-1.78	✓	✓	-
359.0972	3.2	Syringic acid glucosyl ester	182,138	$C_{15}H_{20}O_{10}$	359.0984	-3.82	✓	✓	-
325.0916	4.02	*trans*-*p*-Coumaric acid glucoside	119	$C_{15}H_{18}O_{8}$	325.0929	-2.74	✓	✓	-
311.0396	2.13	*trans*-Caftaric acid	135	$C_{13}H_{12}O_{9}$	311.0409	-2.75	✓	✓	-
447.0913	5.69	Quercetin-3-*O*-rhamnoside	300, 301	$C_{21}H_{20}O_{11}$	447.0933	-2.87	✓	✓	-
315.0710	1.76	Gentisic acid glucoside	108, 152	$C_{13}H_{16}O_{9}$	315.0716	1.90	✓	✓	-
477.1026	5.79	Isorhamnetin-3-*O*-glucoside	314	$C_{22}H_{22}O_{12}$	477.1038	-2.62	✓	✓	✓
639.1547	5.2	Isorhamnetin3,4-diglucoside	477, 315	$C_{28}H_{32}O_{17}$	639.1567	-2.62	✓	✓	✓
433.0755	5.57	Quercetin-3-*O*-arabinoside	300	$C_{20}H_{18}O_{11}$	433.0776	-1.47	✓	✓	-
549.0869	5.52	Quercetin 3-(6''-malonyl-glucoside)	505, 463, 300, 301	$C_{24}H_{22}O_{15}$	549.0886	-2.90	✓	✓	-
461.1075	6.1	Isorhamnetin-3-*O*-rhamnoside	314, 315	$C_{22}H_{22}O_{11}$	461.1089	-2.03	✓	✓	-
295.0445	4.36	Coutaric acid	119	$C_{13}H_{12}O_{8}$	295.0459	-4.75	✓	✓	-
331.0660	1.43	Galloyl glucose	125	$C_{13}H_{16}O_{10}$	331.067070	-3.23	✓	✓	-
561.1382	4.46	Epicatechin epiafzelechin	289	$C_{30}H_{26}O_{11}$	561.1402	-3.98	✓	✓	-
667.1496	5.17	Quercetin-3-*O*-acetyldiglucopyranoside	505, 301	$C_{29}H_{32}O_{18}$	667.1516	-2.38	✓	✓	-

✓- present; - absent

Table S5. Summary of IC_{50} values and viability endpoints on Vero cells.

**Extract/Control**	**IC50 (μg mL^{-1})**	**Viability at 500 μg mL^{-1} (%)**	**Interpretation**
Melphalan	87. 84	5.43	Moderate cytotoxicity
A. senegalensis (MEC1)	>500	55.57	Inactive
P. thonningii (MKC1)	None	105.21	no reduction in viability/non-cytotoxic
